# 6β,15β-Diacet­oxy-1β,7β,13α-trihydr­oxy-7α,20-ep­oxy-*ent*-kaur-16-ene

**DOI:** 10.1107/S1600536810001170

**Published:** 2010-01-16

**Authors:** Xue-Mei Di, Fu-Lin Yan, Chuang Feng, Jian-Min Cui

**Affiliations:** a School of Pharmacy, Xinxiang Medical University, Xinxiang, Henan 453003, People’s Republic of China; b Henan College of Traditional Chinese Medicine, Zhengzhou, Henan 450008, People’s Republic of China

## Abstract

The title compound, C_24_H_34_O_8_, a natural *ent*-kaurane diterpenoid, is composed of four rings with the expected *cis* and *trans* junctions. The crystal structure is stabilized by inter­molecular O—H⋯O hydrogen bonds. In addition, an intra­molecular O—H⋯O hydrogen bond occurs.

## Related literature

For the genus Isodon and diterpenoids, see: Sun *et al.* (2001 ▶); Jung *et al.* (1990 ▶); Li & Tian (2001 ▶); Yan *et al.* (2008 ▶); Han *et al.* (2005 ▶). For bond-length data, see: Allen *et al.* (1987 ▶)
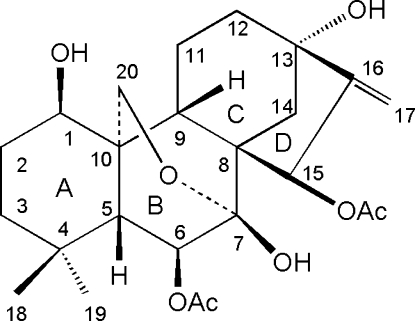


## Experimental

### 

#### Crystal data

C_24_H_34_O_8_
*M* *_r_* = 450.51
Orthorhombic, 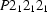

*a* = 10.295 (2) Å
*b* = 13.696 (3) Å
*c* = 15.802 (3) Å
*V* = 2228.1 (8) Å^3^
*Z* = 4
Mo *K*α radiation
μ = 0.10 mm^−1^
*T* = 93 K
0.33 × 0.33 × 0.30 mm

#### Data collection

Rigaku SPIDER diffractometer
18422 measured reflections
2878 independent reflections
2819 reflections with *I* > 2σ(*I*)
*R*_int_ = 0.034
Standard reflections: 0

#### Refinement

*R*[*F* ^2^ > 2σ(*F* ^2^)] = 0.030
*wR*(*F* ^2^) = 0.073
*S* = 1.07
2878 reflections
306 parameters
H atoms treated by a mixture of independent and constrained refinement
Δρ_max_ = 0.21 e Å^−3^
Δρ_min_ = −0.15 e Å^−3^

### 

Data collection: *RAPID-AUTO* (Rigaku, 2004 ▶); cell refinement: *RAPID-AUTO*; data reduction: *RAPID-AUTO*; program(s) used to solve structure: *SHELXS97* (Sheldrick, 2008 ▶); program(s) used to refine structure: *SHELXL97* (Sheldrick, 2008 ▶); molecular graphics: *ORTEP-3* (Farrugia, 1997 ▶) and *DIAMOND* (Brandenburg, 1998 ▶); software used to prepare material for publication: *SHELXTL* (Sheldrick, 2008 ▶).

## Supplementary Material

Crystal structure: contains datablocks global, I. DOI: 10.1107/S1600536810001170/lx2122sup1.cif

Structure factors: contains datablocks I. DOI: 10.1107/S1600536810001170/lx2122Isup2.hkl

Additional supplementary materials:  crystallographic information; 3D view; checkCIF report

## Figures and Tables

**Table 1:** Hydrogen-bond geometry (Å, °)

*D*—H⋯*A*	*D*—H	H⋯*A*	*D*⋯*A*	*D*—H⋯*A*
O2—H2*O*⋯O6^i^	0.85 (3)	1.96 (3)	2.7811 (17)	163 (3)
O5—H5*O*⋯O4	0.88 (3)	2.19 (3)	2.9373 (18)	142 (2)
O6—H6*O*⋯O8^ii^	0.94 (3)	1.83 (3)	2.7600 (17)	170 (2)

Symmetry codes: (i) 
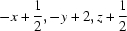
; (ii) 
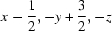
.

**Fig. 1.**
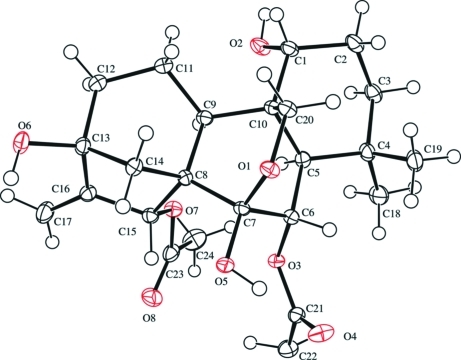
The molecular structure of the title compound with the atom numbering scheme. Displacement ellipsoids are drawn at the 50% probability level. H atoms are presented as a small spheres of arbitrary radius.

**Fig. 2.**
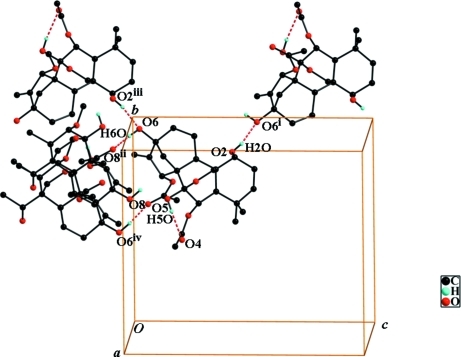
O–H···O interactions (dotted lines) in the structure of the title compound. [Symmetry codes: (i) - x + 1/2, - y + 2, z + 1/2; (ii) x - 1/2, - y + 3/2, - z; (iii) - x + 1/2, - y + 2, z - 1/2; (iv) x + 1/2, - y + 3/2, - z.]

## supplementary crystallographic information

### Comment

The title compound (I), 6β, 15β-Diacetoxy-1β, 7β, 13α-trihydroxy-7α, 20-epoxy-*ent*-kaur-16-ene is a new natural *ent*-kaurane diterpenoid isolated from the medicinal plant *Isodon japonica*. The leaves of this plant has been used as an antibacterial, anti-inflammatory, stomachic, and anthelmintic agent in China, Korean and Japan by local people (Jung *et al.*, 1990; Li & Tian, 2001). The structure of compound (I) was postulated from spectroscopic methods. In order to further confirm the structure and conformation of (I), a crystal structure analysis has been undertaken. The X-ray crystallographic analysis of (I) confirms the molecular structure of (I) proposed by spectroscopic methods.

Fig.1 shows its conformation: three hydroxyl groups adopt β, β, α-orientations at C1, C7 and C13, two acetoxyl groups adopt β-orientations at C6 and C15 respectively. There is a *trans* junction between ring *A* (C1–C5/C10) and ring *B* (C5–C10); *cis* junctions are present between ring *B* and ring *C* (C8/C9/C11–C14), and ring *C* and ring *D* (C8/C13–C16). The bond lengths and angles are within expected aranges (Allen *et al.*, 1987). Conformation of ring can be seen according to the X-ray diffraction pattern (Fig.1). Ring *A* adopts chair conformation, with an average torsion angles of 51.43 (18) °. Rings *B* and *C* adopt boat conformation because of the formation of the oxygen bridge at C-7 and C-20. Ring *D* shows an evenlope conformation; the flap atom, C14, lies 0.693 Å from the plane defined by atoms C8, C15, C16 and C13. In addition, the six-membered rings O1/C20/C10/C5–C7 and O1/C7–C10/C20 both adopt boat conformations. Compound (I) contains nine chiral centers at C1(*R*), C5(*R*), C6(*S*), C7(*S*), C8(*S*), C9(*S*), C10(*S*), C13(*S*) and C15(*R*). Although the absolute configuration could not be reliably determined from anomalous dispersion effects, the negative optical rotation showed this compound to be in the *ent*-kaurane seuies as reported in genus *Isodon* (Sun *et al.*,2001), rather than in the kaurane series, and so allowed us to assign the correct configuration.

The molecular packing (Fig. 2) is stabilized by two different intermolecular O–H···O hydrogen bonds (Table 1; symmetry code as in Fig. 2). The crystal packing (Fig. 2) is further stabilized by an intramolecular O–H···O hydrogen bond (Table 1; symmetry code as in Fig. 2).

### Experimental

The dried and crushed leaves of *Isodon japonica* (17 kg, collected from Tongbai Prefecture, Henan Province, China) were extracted four times with Me_2_CO/H_2_O (7:3, *v*/*v*) at room temperature over a period of six days. The extract was filtered and the solvent was removed under reduced pressure. The residue was then partitioned between water and AcOEt. After removal of the solvent, the AcOEt residue was separated by repeated silica gel (200-300 mesh) column chromatography and recrystallization from CHCl_3_/CH_3_OH(10:1), giving 70 mg of compound (I) (m.p. 505-507 K. Optical rotation: [α]_D_^20^ -79.6 ° (c 0.45, CH_3_OH). Single crystals suitable for X-ray diffraction were obtained by slow evaporation of a solution of the title compound in methanol at room temperature.

### Refinement

All the Friedel pairs were merged. All H atoms were included in calculated positions and refined as riding atoms, with C–H = 0.98Å (CH_3_), 0.99Å (CH_2_), 1.00Å (CH), and O–H = 0.89Å, and with *U*_iso_(H) = 1.2 *U*_eq_(C). The choice of enantiomer was based on comparison of the optical rotation with that of related compounds with known stereochemistry.

### Crystal data

**Table:**

C_24_H_34_O_8_	*F*(000) = 968
*M**_r_* = 450.51	*D*_x_ = 1.343 Mg m^−^^3^
Orthorhombic, *P*2_1_2_1_2_1_	Mo *K*α radiation, λ = 0.71073 Å
Hall symbol: P 2ac 2ab	Cell parameters from 8346 reflections
*a* = 10.295 (2) Å	θ = 3.2–27.5°
*b* = 13.696 (3) Å	µ = 0.10 mm^−^^1^
*c* = 15.802 (3) Å	*T* = 93 K
*V* = 2228.1 (8) Å^3^	Block, colorless
*Z* = 4	0.33 × 0.33 × 0.30 mm

### Data collection

**Table:**

Rigaku SPIDER diffractometer	2819 reflections with *I* > 2σ(*I*)
Radiation source: fine-focus sealed tube	*R*_int_ = 0.034
graphite	θ_max_ = 27.5°, θ_min_ = 3.2°
ω scans	*h* = −13→11
18422 measured reflections	*k* = −17→17
2878 independent reflections	*l* = −20→20

### Refinement

**Table:**

Refinement on *F*^2^	Secondary atom site location: difference Fourier map
Least-squares matrix: full	Hydrogen site location: difference Fourier map
*R*[*F*^2^ > 2σ(*F*^2^)] = 0.030	H atoms treated by a mixture of independent and constrained refinement
*wR*(*F*^2^) = 0.073	*w* = 1/[σ^2^(*F*_o_^2^) + (0.0418*P*)^2^ + 0.1265*P*] where *P* = (*F*_o_^2^ + 2*F*_c_^2^)/3
*S* = 1.07	(Δ/σ)_max_ = 0.001
2878 reflections	Δρ_max_ = 0.21 e Å^−^^3^
306 parameters	Δρ_min_ = −0.15 e Å^−^^3^
0 restraints	Extinction correction: *SHELXL97* (Sheldrick, 2008), Fc^*^=kFc[1+0.001xFc^2^λ^3^/sin(2θ)]^-1/4^
Primary atom site location: structure-invariant direct methods	Extinction coefficient: 0.0063 (13)

### Special details

**Table:**

Geometry. All esds (except the esd in the dihedral angle between two l.s. planes) are estimated using the full covariance matrix. The cell esds are taken into account individually in the estimation of esds in distances, angles and torsion angles; correlations between esds in cell parameters are only used when they are defined by crystal symmetry. An approximate (isotropic) treatment of cell esds is used for estimating esds involving l.s. planes.
Refinement. Refinement of *F*^2^ against ALL reflections. The weighted *R*-factor *wR* and goodness of fit *S* are based on *F*^2^, conventional *R*-factors *R* are based on *F*, with *F* set to zero for negative *F*^2^. The threshold expression of *F*^2^ > 2σ(*F*^2^) is used only for calculating *R*-factors(gt) etc. and is not relevant to the choice of reflections for refinement. *R*-factors based on *F*^2^ are statistically about twice as large as those based on *F*, and *R*-factors based on ALL data will be even larger.

### Fractional atomic coordinates and isotropic or equivalent isotropic displacement parameters (Å^2^)

**Table:**

	*x*	*y*	*z*	*U*_iso_*/*U*_eq_	
O1	0.01173 (10)	0.68667 (8)	0.26057 (7)	0.0149 (2)	
O2	0.30874 (12)	0.88041 (9)	0.42632 (7)	0.0212 (3)	
H2O	0.315 (2)	0.922 (2)	0.4660 (19)	0.058 (8)*	
O3	0.33002 (10)	0.57116 (8)	0.24245 (7)	0.0148 (2)	
O4	0.21962 (12)	0.43421 (9)	0.20969 (8)	0.0253 (3)	
O5	0.10209 (11)	0.61497 (8)	0.14532 (7)	0.0159 (2)	
H5O	0.099 (2)	0.554 (2)	0.1618 (16)	0.050 (7)*	
O6	0.13739 (12)	0.96328 (8)	0.03305 (7)	0.0189 (3)	
H6O	0.105 (2)	0.923 (2)	−0.0103 (16)	0.055 (8)*	
O7	0.43032 (11)	0.75344 (8)	0.18270 (7)	0.0161 (2)	
O8	0.51871 (12)	0.65725 (10)	0.08362 (8)	0.0253 (3)	
C1	0.18434 (16)	0.83486 (12)	0.43078 (10)	0.0161 (3)	
H1	0.1156	0.8864	0.4312	0.019*	
C2	0.17508 (17)	0.77546 (13)	0.51205 (10)	0.0188 (4)	
H2A	0.1894	0.8187	0.5613	0.023*	
H2B	0.0870	0.7470	0.5170	0.023*	
C3	0.27532 (17)	0.69440 (12)	0.51243 (10)	0.0193 (4)	
H3A	0.2716	0.6601	0.5675	0.023*	
H3B	0.3629	0.7235	0.5068	0.023*	
C4	0.25500 (16)	0.62004 (12)	0.44115 (10)	0.0171 (3)	
C5	0.25206 (15)	0.67689 (11)	0.35551 (9)	0.0135 (3)	
H5	0.3437	0.6976	0.3445	0.016*	
C6	0.21348 (15)	0.61224 (11)	0.28031 (9)	0.0134 (3)	
H6	0.1594	0.5571	0.3023	0.016*	
C7	0.13183 (15)	0.66985 (11)	0.21737 (9)	0.0127 (3)	
C8	0.18916 (15)	0.76762 (11)	0.19036 (9)	0.0124 (3)	
C9	0.21621 (16)	0.82954 (11)	0.27169 (10)	0.0144 (3)	
H9	0.3127	0.8358	0.2769	0.017*	
C10	0.16867 (15)	0.77195 (11)	0.35071 (9)	0.0140 (3)	
C11	0.16190 (17)	0.93342 (12)	0.26232 (10)	0.0190 (4)	
H11A	0.0659	0.9314	0.2661	0.023*	
H11B	0.1945	0.9746	0.3092	0.023*	
C12	0.20189 (18)	0.97887 (11)	0.17758 (10)	0.0181 (3)	
H12A	0.2941	0.9992	0.1807	0.022*	
H12B	0.1489	1.0380	0.1673	0.022*	
C13	0.18435 (16)	0.90739 (11)	0.10258 (10)	0.0148 (3)	
C14	0.09702 (16)	0.82277 (11)	0.12983 (10)	0.0148 (3)	
H14A	0.0712	0.7817	0.0811	0.018*	
H14B	0.0182	0.8462	0.1595	0.018*	
C15	0.31094 (15)	0.76055 (11)	0.13340 (9)	0.0140 (3)	
H15	0.3032	0.7026	0.0952	0.017*	
C16	0.30844 (16)	0.85321 (11)	0.08147 (10)	0.0153 (3)	
C17	0.39369 (18)	0.87883 (13)	0.02315 (11)	0.0245 (4)	
H17A	0.4650	0.8372	0.0106	0.029*	
H17B	0.3838	0.9389	−0.0063	0.029*	
C18	0.37174 (18)	0.55017 (14)	0.43998 (11)	0.0238 (4)	
H18A	0.4503	0.5864	0.4245	0.029*	
H18B	0.3563	0.4983	0.3985	0.029*	
H18C	0.3830	0.5213	0.4962	0.029*	
C19	0.13432 (17)	0.55891 (13)	0.45997 (10)	0.0225 (4)	
H19A	0.1462	0.5241	0.5136	0.027*	
H19B	0.1209	0.5116	0.4143	0.027*	
H19C	0.0584	0.6018	0.4642	0.027*	
C20	0.02593 (15)	0.74576 (12)	0.33582 (9)	0.0159 (3)	
H20A	−0.0079	0.7095	0.3853	0.019*	
H20B	−0.0256	0.8063	0.3296	0.019*	
C21	0.32071 (17)	0.47860 (12)	0.21399 (10)	0.0176 (3)	
C22	0.45078 (17)	0.43876 (13)	0.19149 (12)	0.0228 (4)	
H22A	0.4994	0.4244	0.2433	0.027*	
H22B	0.4985	0.4870	0.1578	0.027*	
H22C	0.4401	0.3787	0.1586	0.027*	
C23	0.52622 (16)	0.69893 (12)	0.15079 (11)	0.0195 (3)	
C24	0.64137 (18)	0.69693 (15)	0.20862 (13)	0.0302 (4)	
H24A	0.7075	0.6530	0.1854	0.036*	
H24B	0.6145	0.6736	0.2646	0.036*	
H24C	0.6775	0.7629	0.2137	0.036*	

### Atomic displacement parameters (Å^2^)

**Table:**

	*U*^11^	*U*^22^	*U*^33^	*U*^12^	*U*^13^	*U*^23^
O1	0.0130 (5)	0.0174 (5)	0.0142 (5)	−0.0016 (4)	0.0013 (4)	−0.0031 (4)
O2	0.0245 (7)	0.0228 (6)	0.0164 (6)	−0.0091 (5)	0.0037 (5)	−0.0077 (5)
O3	0.0171 (6)	0.0124 (5)	0.0148 (5)	0.0013 (4)	0.0024 (4)	−0.0021 (4)
O4	0.0259 (7)	0.0175 (6)	0.0323 (7)	−0.0004 (5)	−0.0010 (6)	−0.0081 (5)
O5	0.0234 (6)	0.0126 (5)	0.0117 (5)	−0.0015 (5)	−0.0025 (5)	−0.0025 (4)
O6	0.0235 (6)	0.0154 (6)	0.0178 (5)	−0.0023 (5)	−0.0055 (5)	0.0056 (5)
O7	0.0139 (6)	0.0172 (5)	0.0174 (5)	0.0017 (5)	−0.0008 (4)	−0.0015 (5)
O8	0.0275 (7)	0.0266 (7)	0.0218 (6)	0.0078 (6)	0.0066 (5)	−0.0018 (5)
C1	0.0178 (8)	0.0168 (7)	0.0138 (7)	−0.0032 (6)	0.0028 (6)	−0.0041 (6)
C2	0.0210 (9)	0.0236 (8)	0.0119 (7)	−0.0040 (7)	0.0017 (6)	−0.0034 (6)
C3	0.0213 (9)	0.0248 (9)	0.0117 (7)	−0.0028 (7)	−0.0011 (6)	−0.0005 (6)
C4	0.0202 (9)	0.0191 (8)	0.0121 (7)	−0.0011 (7)	−0.0010 (6)	0.0015 (6)
C5	0.0131 (8)	0.0149 (7)	0.0125 (7)	−0.0016 (6)	−0.0011 (6)	0.0003 (6)
C6	0.0147 (8)	0.0132 (7)	0.0124 (7)	0.0004 (6)	0.0020 (6)	−0.0010 (6)
C7	0.0153 (8)	0.0122 (7)	0.0107 (6)	−0.0013 (6)	0.0001 (6)	−0.0025 (6)
C8	0.0137 (8)	0.0124 (7)	0.0111 (6)	−0.0001 (6)	−0.0003 (6)	0.0003 (5)
C9	0.0167 (8)	0.0131 (7)	0.0133 (7)	−0.0008 (6)	0.0018 (6)	−0.0020 (6)
C10	0.0169 (8)	0.0131 (7)	0.0120 (7)	−0.0008 (6)	0.0019 (6)	−0.0023 (6)
C11	0.0269 (9)	0.0129 (7)	0.0172 (7)	0.0015 (7)	0.0025 (7)	−0.0020 (6)
C12	0.0239 (9)	0.0123 (7)	0.0182 (7)	0.0001 (7)	0.0004 (7)	−0.0006 (6)
C13	0.0173 (8)	0.0133 (7)	0.0137 (7)	−0.0001 (6)	−0.0022 (6)	0.0018 (6)
C14	0.0151 (8)	0.0137 (7)	0.0156 (7)	0.0007 (6)	−0.0009 (6)	0.0013 (6)
C15	0.0149 (8)	0.0140 (7)	0.0130 (7)	−0.0007 (6)	−0.0005 (6)	−0.0013 (6)
C16	0.0174 (8)	0.0130 (7)	0.0153 (7)	−0.0014 (6)	−0.0006 (6)	−0.0001 (6)
C17	0.0265 (10)	0.0186 (8)	0.0283 (9)	0.0022 (7)	0.0086 (8)	0.0072 (7)
C18	0.0291 (10)	0.0249 (9)	0.0172 (8)	0.0059 (8)	−0.0038 (7)	0.0020 (7)
C19	0.0296 (10)	0.0230 (8)	0.0148 (8)	−0.0056 (8)	0.0014 (7)	0.0032 (7)
C20	0.0174 (8)	0.0174 (7)	0.0129 (7)	−0.0001 (7)	0.0004 (6)	−0.0028 (6)
C21	0.0258 (9)	0.0134 (7)	0.0135 (7)	0.0038 (7)	−0.0019 (7)	0.0001 (6)
C22	0.0262 (10)	0.0188 (8)	0.0233 (8)	0.0048 (7)	0.0012 (7)	−0.0017 (7)
C23	0.0183 (8)	0.0154 (8)	0.0249 (8)	0.0012 (7)	0.0066 (7)	0.0031 (6)
C24	0.0222 (10)	0.0273 (10)	0.0410 (11)	0.0065 (8)	−0.0040 (8)	−0.0005 (8)

### Geometric parameters (Å, °)

**Table:**

O1—C7	1.431 (2)	C9—C11	1.536 (2)
O1—C20	1.446 (2)	C9—C10	1.556 (2)
O2—C1	1.426 (2)	C9—H9	1.0000
O2—H2O	0.85 (3)	C10—C20	1.531 (2)
O3—C21	1.349 (2)	C11—C12	1.533 (2)
O3—C6	1.454 (2)	C11—H11A	0.9900
O4—C21	1.207 (2)	C11—H11B	0.9900
O5—C7	1.398 (2)	C12—C13	1.548 (2)
O5—H5O	0.88 (3)	C12—H12A	0.9900
O6—C13	1.424 (2)	C12—H12B	0.9900
O6—H6O	0.94 (3)	C13—C16	1.515 (2)
O7—C23	1.337 (2)	C13—C14	1.529 (2)
O7—C15	1.458 (2)	C14—H14A	0.9900
O8—C23	1.208 (2)	C14—H14B	0.9900
C1—C2	1.523 (2)	C15—C16	1.511 (2)
C1—C10	1.539 (2)	C15—H15	1.0000
C1—H1	1.0000	C16—C17	1.320 (2)
C2—C3	1.516 (2)	C17—H17A	0.9500
C2—H2A	0.9900	C17—H17B	0.9500
C2—H2B	0.9900	C18—H18A	0.9800
C3—C4	1.533 (2)	C18—H18B	0.9800
C3—H3A	0.9900	C18—H18C	0.9800
C3—H3B	0.9900	C19—H19A	0.9800
C4—C19	1.527 (2)	C19—H19B	0.9800
C4—C18	1.536 (2)	C19—H19C	0.9800
C4—C5	1.562 (2)	C20—H20A	0.9900
C5—C6	1.534 (2)	C20—H20B	0.9900
C5—C10	1.561 (2)	C21—C22	1.489 (2)
C5—H5	1.0000	C22—H22A	0.9800
C6—C7	1.523 (2)	C22—H22B	0.9800
C6—H6	1.0000	C22—H22C	0.9800
C7—C8	1.524 (2)	C23—C24	1.497 (3)
C8—C14	1.544 (2)	C24—H24A	0.9800
C8—C15	1.546 (2)	C24—H24B	0.9800
C8—C9	1.565 (2)	C24—H24C	0.9800
			
C7—O1—C20	113.28 (11)	C12—C11—H11B	109.4
C1—O2—H2O	108.9 (18)	C9—C11—H11B	109.4
C21—O3—C6	116.25 (13)	H11A—C11—H11B	108.0
C7—O5—H5O	106.4 (16)	C11—C12—C13	112.37 (13)
C13—O6—H6O	111.6 (16)	C11—C12—H12A	109.1
C23—O7—C15	117.28 (12)	C13—C12—H12A	109.1
O2—C1—C2	109.35 (14)	C11—C12—H12B	109.1
O2—C1—C10	107.36 (12)	C13—C12—H12B	109.1
C2—C1—C10	112.80 (13)	H12A—C12—H12B	107.9
O2—C1—H1	109.1	O6—C13—C16	112.32 (13)
C2—C1—H1	109.1	O6—C13—C14	115.17 (13)
C10—C1—H1	109.1	C16—C13—C14	100.76 (12)
C3—C2—C1	110.61 (13)	O6—C13—C12	106.89 (12)
C3—C2—H2A	109.5	C16—C13—C12	112.34 (13)
C1—C2—H2A	109.5	C14—C13—C12	109.42 (13)
C3—C2—H2B	109.5	C13—C14—C8	100.61 (12)
C1—C2—H2B	109.5	C13—C14—H14A	111.7
H2A—C2—H2B	108.1	C8—C14—H14A	111.7
C2—C3—C4	113.00 (13)	C13—C14—H14B	111.7
C2—C3—H3A	109.0	C8—C14—H14B	111.7
C4—C3—H3A	109.0	H14A—C14—H14B	109.4
C2—C3—H3B	109.0	O7—C15—C16	111.13 (12)
C4—C3—H3B	109.0	O7—C15—C8	112.12 (11)
H3A—C3—H3B	107.8	C16—C15—C8	104.46 (12)
C19—C4—C3	109.39 (14)	O7—C15—H15	109.7
C19—C4—C18	107.29 (14)	C16—C15—H15	109.7
C3—C4—C18	108.41 (14)	C8—C15—H15	109.7
C19—C4—C5	115.24 (13)	C17—C16—C15	126.22 (15)
C3—C4—C5	107.95 (13)	C17—C16—C13	125.76 (15)
C18—C4—C5	108.38 (13)	C15—C16—C13	107.82 (13)
C6—C5—C4	112.86 (12)	C16—C17—H17A	120.0
C6—C5—C10	107.54 (12)	C16—C17—H17B	120.0
C4—C5—C10	117.94 (13)	H17A—C17—H17B	120.0
C6—C5—H5	105.9	C4—C18—H18A	109.5
C4—C5—H5	105.9	C4—C18—H18B	109.5
C10—C5—H5	105.9	H18A—C18—H18B	109.5
O3—C6—C7	112.79 (12)	C4—C18—H18C	109.5
O3—C6—C5	109.17 (12)	H18A—C18—H18C	109.5
C7—C6—C5	110.47 (12)	H18B—C18—H18C	109.5
O3—C6—H6	108.1	C4—C19—H19A	109.5
C7—C6—H6	108.1	C4—C19—H19B	109.5
C5—C6—H6	108.1	H19A—C19—H19B	109.5
O5—C7—O1	106.61 (12)	C4—C19—H19C	109.5
O5—C7—C6	111.98 (13)	H19A—C19—H19C	109.5
O1—C7—C6	104.41 (12)	H19B—C19—H19C	109.5
O5—C7—C8	109.21 (12)	O1—C20—C10	110.77 (12)
O1—C7—C8	109.07 (12)	O1—C20—H20A	109.5
C6—C7—C8	115.11 (13)	C10—C20—H20A	109.5
C7—C8—C14	111.42 (13)	O1—C20—H20B	109.5
C7—C8—C15	114.96 (12)	C10—C20—H20B	109.5
C14—C8—C15	99.67 (11)	H20A—C20—H20B	108.1
C7—C8—C9	108.37 (12)	O4—C21—O3	123.60 (15)
C14—C8—C9	110.66 (12)	O4—C21—C22	125.26 (15)
C15—C8—C9	111.57 (12)	O3—C21—C22	111.11 (14)
C11—C9—C10	115.62 (13)	C21—C22—H22A	109.5
C11—C9—C8	110.98 (13)	C21—C22—H22B	109.5
C10—C9—C8	109.17 (12)	H22A—C22—H22B	109.5
C11—C9—H9	106.9	C21—C22—H22C	109.5
C10—C9—H9	106.9	H22A—C22—H22C	109.5
C8—C9—H9	106.9	H22B—C22—H22C	109.5
C20—C10—C1	110.99 (12)	O8—C23—O7	123.25 (16)
C20—C10—C9	107.30 (13)	O8—C23—C24	125.35 (16)
C1—C10—C9	110.06 (12)	O7—C23—C24	111.40 (15)
C20—C10—C5	109.87 (12)	C23—C24—H24A	109.5
C1—C10—C5	111.67 (13)	C23—C24—H24B	109.5
C9—C10—C5	106.78 (12)	H24A—C24—H24B	109.5
C12—C11—C9	111.26 (13)	C23—C24—H24C	109.5
C12—C11—H11A	109.4	H24A—C24—H24C	109.5
C9—C11—H11A	109.4	H24B—C24—H24C	109.5
			
O2—C1—C2—C3	−62.53 (16)	C8—C9—C10—C20	−54.65 (15)
C10—C1—C2—C3	56.86 (18)	C11—C9—C10—C1	−49.55 (18)
C1—C2—C3—C4	−62.82 (18)	C8—C9—C10—C1	−175.51 (13)
C2—C3—C4—C19	−71.09 (17)	C11—C9—C10—C5	−170.93 (13)
C2—C3—C4—C18	172.21 (14)	C8—C9—C10—C5	63.11 (15)
C2—C3—C4—C5	55.00 (18)	C6—C5—C10—C20	47.55 (15)
C19—C4—C5—C6	−49.47 (19)	C4—C5—C10—C20	−81.40 (16)
C3—C4—C5—C6	−172.06 (13)	C6—C5—C10—C1	171.15 (12)
C18—C4—C5—C6	70.72 (17)	C4—C5—C10—C1	42.20 (18)
C19—C4—C5—C10	76.95 (18)	C6—C5—C10—C9	−68.50 (15)
C3—C4—C5—C10	−45.64 (18)	C4—C5—C10—C9	162.55 (13)
C18—C4—C5—C10	−162.86 (14)	C10—C9—C11—C12	−173.29 (14)
C21—O3—C6—C7	−94.07 (15)	C8—C9—C11—C12	−48.25 (18)
C21—O3—C6—C5	142.72 (13)	C9—C11—C12—C13	45.16 (19)
C4—C5—C6—O3	−91.32 (15)	C11—C12—C13—O6	141.31 (14)
C10—C5—C6—O3	136.89 (12)	C11—C12—C13—C16	−95.05 (16)
C4—C5—C6—C7	144.10 (13)	C11—C12—C13—C14	15.98 (19)
C10—C5—C6—C7	12.31 (16)	O6—C13—C14—C8	168.34 (12)
C20—O1—C7—O5	−178.14 (11)	C16—C13—C14—C8	47.24 (14)
C20—O1—C7—C6	63.19 (15)	C12—C13—C14—C8	−71.27 (15)
C20—O1—C7—C8	−60.34 (15)	C7—C8—C14—C13	−171.67 (12)
O3—C6—C7—O5	53.61 (16)	C15—C8—C14—C13	−49.89 (13)
C5—C6—C7—O5	176.09 (12)	C9—C8—C14—C13	67.68 (15)
O3—C6—C7—O1	168.56 (11)	C23—O7—C15—C16	−97.73 (16)
C5—C6—C7—O1	−68.96 (15)	C23—O7—C15—C8	145.78 (13)
O3—C6—C7—C8	−71.91 (16)	C7—C8—C15—O7	−87.21 (15)
C5—C6—C7—C8	50.57 (17)	C14—C8—C15—O7	153.57 (12)
O5—C7—C8—C14	55.65 (16)	C9—C8—C15—O7	36.68 (16)
O1—C7—C8—C14	−60.50 (15)	C7—C8—C15—C16	152.35 (13)
C6—C7—C8—C14	−177.42 (12)	C14—C8—C15—C16	33.14 (14)
O5—C7—C8—C15	−56.79 (17)	C9—C8—C15—C16	−83.76 (14)
O1—C7—C8—C15	−172.94 (11)	O7—C15—C16—C17	59.4 (2)
C6—C7—C8—C15	70.14 (17)	C8—C15—C16—C17	−179.46 (16)
O5—C7—C8—C9	177.63 (12)	O7—C15—C16—C13	−125.49 (13)
O1—C7—C8—C9	61.48 (15)	C8—C15—C16—C13	−4.39 (15)
C6—C7—C8—C9	−55.43 (16)	O6—C13—C16—C17	25.4 (2)
C7—C8—C9—C11	−131.48 (14)	C14—C13—C16—C17	148.54 (17)
C14—C8—C9—C11	−9.03 (17)	C12—C13—C16—C17	−95.1 (2)
C15—C8—C9—C11	100.98 (15)	O6—C13—C16—C15	−149.65 (13)
C7—C8—C9—C10	−2.89 (17)	C14—C13—C16—C15	−26.56 (15)
C14—C8—C9—C10	119.55 (14)	C12—C13—C16—C15	89.81 (15)
C15—C8—C9—C10	−130.43 (13)	C7—O1—C20—C10	−1.88 (17)
O2—C1—C10—C20	−162.52 (13)	C1—C10—C20—O1	−179.99 (12)
C2—C1—C10—C20	76.95 (17)	C9—C10—C20—O1	59.73 (16)
O2—C1—C10—C9	−43.89 (17)	C5—C10—C20—O1	−56.00 (16)
C2—C1—C10—C9	−164.43 (14)	C6—O3—C21—O4	8.8 (2)
O2—C1—C10—C5	74.52 (15)	C6—O3—C21—C22	−169.57 (13)
C2—C1—C10—C5	−46.02 (18)	C15—O7—C23—O8	1.0 (2)
C11—C9—C10—C20	71.31 (16)	C15—O7—C23—C24	−179.03 (14)

### Hydrogen-bond geometry (Å, °)

**Table:**

*D*—H···*A*	*D*—H	H···*A*	*D*···*A*	*D*—H···*A*
O2—H2O···O6^i^	0.85 (3)	1.96 (3)	2.7811 (17)	163 (3)
O5—H5O···O4	0.88 (3)	2.19 (3)	2.9373 (18)	142 (2)
O6—H6O···O8^ii^	0.94 (3)	1.83 (3)	2.7600 (17)	170 (2)

Symmetry codes: (i) −*x*+1/2, −*y*+2, *z*+1/2; (ii) *x*−1/2, −*y*+3/2, −*z*.
